# A spatial obesity risk score for describing the obesogenic environment using kernel density estimation: development and parameter variation

**DOI:** 10.1186/s12874-023-01883-y

**Published:** 2023-03-17

**Authors:** Maximilian Präger, Christoph Kurz, Rolf Holle, Werner Maier, Michael Laxy

**Affiliations:** 1 Department of Sport and Health Sciences, Technical University of Munich, Munich, Germany; 2 Institute for Medical Information Processing, Biometry, and Epidemiology (IBE), Ludwig-Maximilians-University Munich, Munich, Germany; 3 Munich School of Management and Munich Center of Health Sciences, Ludwig-Maximilians-University Munich, Munich, Germany; 4 German Center for Diabetes Research, Neuherberg, Germany

**Keywords:** Risk score, Kernel density estimation, Obesity, Spatial, OpenStreetMap

## Abstract

**Background:**

Overweight and obesity are severe public health problems worldwide. Obesity can lead to chronic diseases such as type 2 diabetes mellitus. Environmental factors may affect lifestyle aspects and are therefore expected to influence people’s weight status. To assess environmental risks, several methods have been tested using geographic information systems. Freely available data from online geocoding services such as OpenStreetMap (OSM) can be used to determine the spatial distribution of these obesogenic factors. The aim of our study was to develop and test a spatial obesity risk score (SORS) based on data from OSM and using kernel density estimation (KDE).

**Methods:**

Obesity-related factors were downloaded from OSM for two municipalities in Bavaria, Germany. We visualized obesogenic and protective risk factors on maps and tested the spatial heterogeneity via Ripley’s K function. Subsequently, we developed the SORS based on positive and negative KDE surfaces. Risk score values were estimated at 50 random spatial data points. We examined the bandwidth, edge correction, weighting, interpolation method, and numbers of grid points. To account for uncertainty, a spatial bootstrap (1000 samples) was integrated, which was used to evaluate the parameter selection via the ANOVA F statistic.

**Results:**

We found significantly clustered patterns of the obesogenic and protective environmental factors according to Ripley’s K function. Separate density maps enabled ex ante visualization of the positive and negative density layers. Furthermore, visual inspection of the final risk score values made it possible to identify overall high- and low-risk areas within our two study areas. Parameter choice for the bandwidth and the edge correction had the highest impact on the SORS results.

**Discussion:**

The SORS made it possible to visualize risk patterns across our study areas. Our score and parameter testing approach has been proven to be geographically scalable and can be applied to other geographic areas and in other contexts. Parameter choice played a major role in the score results and therefore needs careful consideration in future applications.

**Supplementary Information:**

The online version contains supplementary material available at 10.1186/s12874-023-01883-y.

## Background

Overweight and obesity are severe problems worldwide, causing a number of diseases such as type 2 diabetes, and thus reducing expected life years and quality of life [1, 2]. In Germany, for example, the prevalence of being overweight or obese among adults was 54.0% according to the GEDA (GEDA, German Health Update) study from the Robert Koch Institute (a national public health institute in Germany) in 2014/2015, with men being affected more often than women [3]. Other personal aspects affecting obesity besides gender were low education and higher age according to Mader et al. [4]. In addition, several German cohort studies have shown that the average weight in middle-aged populations increased slightly during recent years [5].

Obesity has become a major public health concern, and recent studies describe regional heterogeneity [6, 7]. In obesity-related research, the term “obesogenic environment” describes environmental influences such as green space or fast food restaurants on the development of obesity [8, 9], which has been investigated intensively in the past [10]. Several approaches have been developed in order to analyze the effect of the environment on the risk of obesity. Examples include obesogeneity assessment via questionnaires [11] and via data visualization tools for obesity policy [12].

Some geographic modeling approaches were used to characterize the accumulation of environmental factors. Common techniques include kernel density estimation (KDE), a density method that allows for the estimation of a continuous risk surface [13, 14], as well as hot spot mapping [15] and further geographic information system (GIS) methods [16]. These methods can be used to develop obesity risk scores [17].

Online geocoding services offer low-cost geographic data for researchers that can be downloaded and used for spatial statistical analyses. Their validity has been investigated in the past with reasonable results regarding completeness of environmental factors and positional accuracy of their coordinates [18, 19]. Therefore, they offer a rich database on which geographic tools can be built. However, geocoding services such as Google Maps offer data only in a limited way. In contrast, geodata from OpenStreetMap (OSM) contain geographic information provided by volunteers and thus are less restricted [20]. In a recent study, we performed an extensive literature search to identify obesity-related environmental factors [18]. Furthermore, we operationalized and downloaded corresponding points of interest (POIs).

The aim of our study extends this approach by developing and testing the spatial obesity risk score (SORS) based on data from OSM. The SORS calculates the obesity risk for each geographic point in a given region based on the local density of positive and/or negative obesity-related environmental factors. In our study, we developed a methodological framework for risk score estimation using KDE and tested the influence of five KDE parameters on the SORS values: (1) bandwidth, (2) edge correction via the size of the download area, (3) number of grid points, (4) risk interpolation method, and (5) weighting scheme of the environmental factors.

## Methods

### Overview of the study approach

**Fig. 1:**
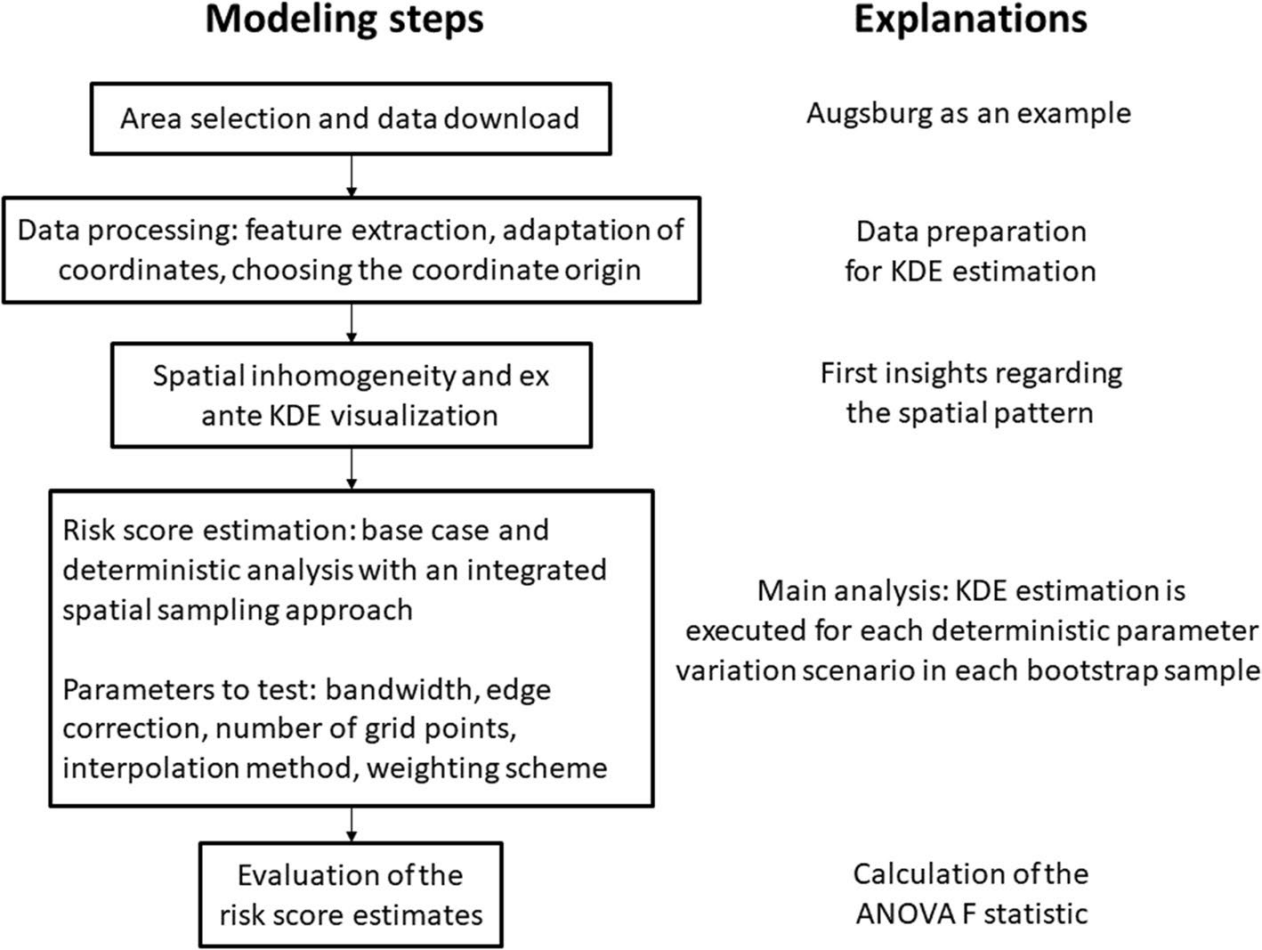
Modelling process for obesity risk score estimation. ANOVA = analysis of variances, KDE = kernel density estimation

We describe the general strategy of obesity risk score estimation, which consists of several steps, by applying it to two regions in Bavaria, Germany. First, we chose our study area and downloaded POIs related to obesogenic environmental factors (cf. [18]). Second, the data were processed to adjust for some imprecisions and minimize redundancy, e.g., we adapted the coordinate units to represent the same length. Third, we analyzed the spatial heterogeneity of the study area and inspected the study area visually to get first insights regarding the distribution of POIs. Fourth, we presented the basic risk score estimation approach, as well as a deterministic sensitivity analysis, in which a spatial POI resampling approach was integrated. This approach made it finally possible in a last step to evaluate the deterministic parameter selection via the ANOVA F statistic. An overview of the risk score estimation process is shown in Fig. 1. Further details regarding each step are given below.

### Area selection and data download

We based our analysis on the overall obesogenic and protective environmental factors identified in our previous study [18]. The list of chosen variables for the score was derived via an extensive literature review, i.e., previous work by Mackenbach et al. [10], Jia et al. [21], and enriched with our own further searches.

We chose two areas in the south of Bavaria, Germany, to illustrate our approach and develop the SORS. Our aim was to base the geographical analysis on different levels of urbanity. The first area was the city of Augsburg with about 300,000 inhabitants covering an area of 147 km^2^. A more rural region, the town of Meitingen with 11,000 inhabitants and a size of 30 km^2^ lying 20 km north of Augsburg, was chosen as the second area. Information on these regions was available from the German Federal Statistical Office [22]. We selected the region around the city of Augsburg, as it is well known within obesity- and diabetes-related research [23].

Spatial POIs related to the selected variables, such as fast food restaurants and parks (compare Additional file 1), were downloaded from OSM using the online data filtering tool Overpass turbo [24]. Data regarding the area borders of Augsburg and Meitingen were downloaded in shape file format from a geographic online portal provided by the Bavarian government [25]. We directly downloaded maps intended for graphical visualization of results from the OSM web page [26]. Additional file 2 contains further information regarding data download.

### Data processing

The downloaded GeoJSON files contained information on each POI regarding name, type of obesity-related environmental factor, spatial coordinates in latitude and longitude format, and other characteristics such as opening hours and street addresses. The relevant information, i.e., name, coordinates, and type of environmental factor, was extracted for each spatial POI and processed into lists and data frames. Table A1 of Additional file 1 gives an overview of the downloaded and processed variables from OSM.

As a further pre-processing step, we introduced a synthetic origin to the south-west of both study areas and adapted the length of a longitudinal coordinate unit to the length of a latitudinal coordinate unit. The schematic structure after the introduction of the origin and the coordinate adaptation is shown in Table 1. The single bus stops were reduced from POIs to centroids of dense regions of bus stops. Further details regarding these additional pre-processing steps can be found within Additional file 2.

**Table 1:** Schematic example of six processed POIs

Categorization			Coordinates^a^	
Type of environmental factor	Category of the POI^b^	Subcategory of the POI^c^	Longitude	Latitude
Obesogenic	Unhealthy_food	Pastry	0.2595	0.3189
Obesogenic	Unhealthy_food	Pastry	0.2488	0.3161
Obesogenic	Unhealthy_food	Sweets	0.2303	0.2657
Protective	Physical_activity	Canoe	0.2789	0.2966
Protective	Physical_activity	Climbing	0.2404	0.2867
Protective	Physical_activity	Climbing	0.3009	0.2084

*POI* Point of interest

^a^Coordinates after equidistant transformation and relative to the synthetic origin

^b^Categories were derived from the literature [10, 18]

^c^The subcategories were derived from OpenStreetMap map features [27]

### Spatial inhomogeneity and ex ante KDE visualization

Ripley’s K function was calculated for the unweighted obesogenic and protective POIs separately to describe the spatial inhomogeneity of the two study areas. The K function is a second-order moment function that is based on the variance of the radial interpoint distance r around each POI [28]. It compares the cumulative number of actual POIs with the number of expected POIs under random distribution assumption [29]. This random comparison process is realized via the Poisson process, which has a K value of r^2^π [30]. K functions lying above the K values of a random process therefore represent clustered patterns, whereas smaller values indicate regular processes [31]. We examined the spatial inhomogeneity to investigate ex ante whether clusters are expected to occur in our subsequent POI analysis. The isotropic edge correction, which is a method based on weighting of the POIs according to the probability of their next neighbors being within the study area, was applied to the K function [32]. In order to test whether the K functions of the POIs were significantly different from the K function of a random point pattern, we estimated bootstrap confidence bands around the K functions of the POIs based on the method of Loh with 1000 simulations [33].

In order to visualize the spatial distribution of the POIs, we created KDEs separately for positive and negative spatial data points [34]. These density layers were superimposed and shown together on a single map. Within this process, we defined certain parameter choices as the base case, which were then changed as part of the sensitivity analysis in a later step.

### Risk score estimation

We estimated the SORS based on the integration of obesogenic and protective kernel densities into a combined score. The aim of KDE is to provide a smooth and continuous estimation of the accumulation of spatial data points based on a sliding window technique. The geographic plane is represented by two dimensions and the estimated densities account for a third dimension, which thus leads to a three-dimensional mountainous structure, a so-called “risk surface”. To visualize this structure on a map, the level of the density coordinate can be plotted by contour lines or via coloring [35]. An overview of this method is provided by Hastie et al., for example, as well as by King et al. [36, 37].

To estimate risk score values, several steps have been performed. First, the positive spatial data points were included into a single positive spatial data layer. Analogously, the negative spatial data points were integrated into a data layer. Second, an observation window together with a grid of suitable size was set up and laid on the respective study area. For each of the following calculations, the same grid was used. Third, KDEs were performed to generate a risk surface based only on positive environmental factors and a second risk surface based only on negative environmental factors. Following this process, a density value based on positive factors and a value based on negative factors were generated at each grid point. Fourth, these positive and negative estimates were set against each other by taking the difference, which results in the final score values at the grid points [34]. Finally, to determine the risk value at the exact desired spatial location, interpolation methods were applied.

### Deterministic analysis

The procedure described above implies several parameter choices within its different steps. We tested alternative values for five of these parameters: bandwidth, edge correction, number of grid points, interpolation method, and an alternative weighting scheme (see overview in Table 2). For each given parameter variation, the remaining KDE parameters were set to their base case values (see also Table 2). Further explanations regarding the parameters are provided below.

**Table 2:** Overview on the sensitivity parameters

Parameter	Base case	Deterministic sensitivity analysis
1) Bandwidth	Method of Terrell [38]	1/3, 2/3, 4/3, 5/3 of the base case bandwidth
2) Size of download area (edge correction)	1.4 * side length of the exact box	x * side length of the exact box, with x ϵ {1.8, 2.2, 2.6, 3.0}
3) Number of grid points	35 × 35 grid points	70 × 70 grid points, 105 × 105 grid points
4) Interpolation method	Automatic interpolation with the R function “interp.surface”	Inverse distance weighting density of the nearest grid cell ordinary Kriging
5) Weighting scheme of the environmental factors	Equal weighting with unity	Double weighting of supermarkets and gyms

#### Bandwidth selection

The bandwidth is an important parameter in KDE that determines the degree of smoothing. An increased bandwidth results in a higher smoothing level. For the base case, the oversmoothing bandwidth proposed by Terrell et al. [38] was chosen, which can be described as the maximum smoothing degree that can be suitably applied to a given data set. Within the deterministic scenarios, alternative higher and lower values in steps of 1/3 of the base case bandwidth were tested. We used a pooled bandwidth as described by Davies and colleagues [39].

#### Size of the download area (edge correction)

Restricting the observational window for KDE to the area boundaries would lead to an underestimation of the true densities at the borders. These effects are especially high if the observation window has a complex structure. To correct for edge effects, we defined a rectangular observation window around the area borders, which simultaneously served as the POI download area and as the KDE area. As a first step, a window was determined from the maximum latitude, maximum longitude, minimum latitude, and minimum longitude of the city boundaries. This minimum bounding rectangle around the study area will be called the “exact box”. For the base case, each side length of the rectangle was increased by 40%, and the resulting rectangle was held in a concentric orientation to the smaller rectangle from the step before. The whole observation window was used for the creation of the risk surface. However, risk score values were only evaluated at locations that lay within the boundaries of the respective study area. To determine the effects of the window size, we gradually increased the download area in steps of 40% of the exact box.

#### Number of grid points

A further central parameter of KDE is the number of grid points. These points were distributed equally on the estimation rectangle. Therefore, KDEs were generated for grid points lying both inside and outside the study area, and both types of grid points were used to estimate the risk score inside the borders of the study areas. A higher number of grid points means that the amount of interpolation is reduced, as more spatial points exist at which an exact estimation is known. To preserve the location of and distance between the inner grid points within the edge correction scenarios, we increased the number of grid points in 40% steps according to the increase in the side length of the download area. Setting the number of grid points to 25 × 25 for the exact box, this led to a base case grid of 35 × 35. For each of the following 40% steps of edge correction, again 10 additional grid points were added to each grid point dimension. Within the remaining grid point sensitivity analyses, increased numbers of 70 × 70 and 105 × 105 grid points were tested.

#### Interpolation method

The automatic interpolation function “interp.surface” of the R package “fields” [40] was applied within the base case. As an alternative interpolation approach, we used inverse distance weighting using the four grid points surrounding a targeted evaluation point. Furthermore, extraction of the score value of the grid point with the minimum distance to the targeted evaluation point was implemented as the third interpolation method [41]. As a fourth scenario, we chose ordinary Kriging which has proven its reliability for interpolating surfaces [42].

#### Weighting scheme of the environmental factors

Several approaches exist for the design of proper weighting schemes. One approach, for example, would be to weight factors according to the strength of evidence for a positive or negative correlation. Regarding our analysis, we chose an equal weighting scheme as a base case scenario. To test an alternative weighting scenario, we followed the approach of Jones-Smith et al. [34]. These authors gave a higher weighting to factors that generally reach more people because of their longer opening hours or size. For the deterministic sensitivity analysis, we therefore tested a double weighting of supermarkets and gyms.

### Resampling approach to account for uncertainty in the distribution of the POIs

Uncertainty concerning POIs was integrated into density score estimations via a spatial bootstrapping method. We generated 1000 bootstrap samples for the positive and 1000 bootstrap samples for the negative environmental POIs at random with replacement. For each of the samples, the parameter variation described above was executed. This made it possible to integrate probabilistic variation of POIs into SORS value estimation for each deterministic scenario. These uncertainty estimates were used for the calculation of the ANOVA F statistic, which we applied to compare the deterministic sensitivity scenarios for a given parameter. We describe further details regarding the sampling process in Additional file 2.

### Evaluation of the risk score estimates

**Fig. 2:**
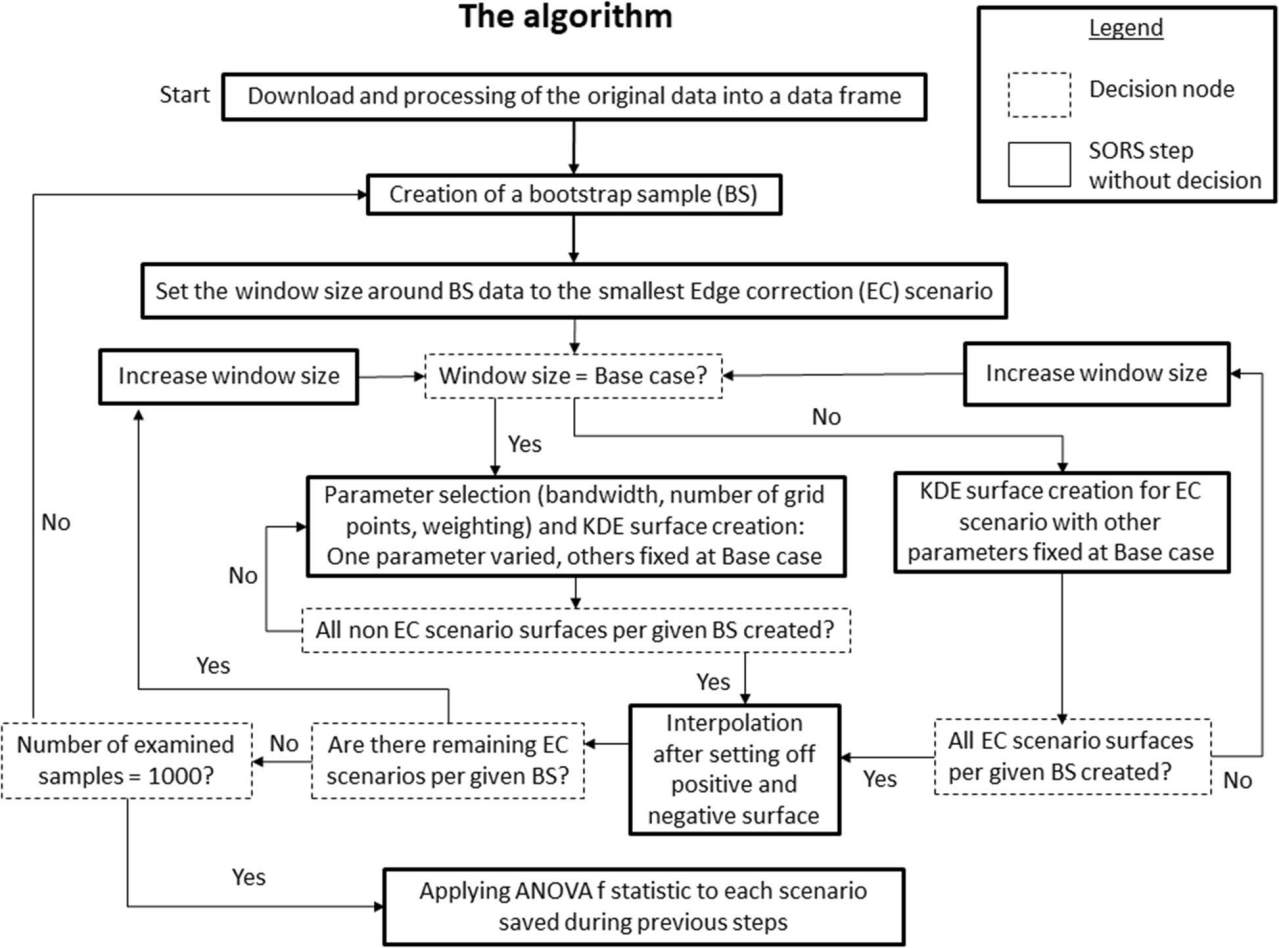
Algorithm describing the combination of deterministic and probabilistic analysis, BS = bootstrap sample, EC = edge correction

A random sample (*N* = 50) of spatial data points (evaluation points, EPs) was drawn from each of the two areas for which we calculated and compared risk score results across the scenarios defined above. Our aim was to develop a robust and stable score that accounts for uncertainty and exhibits discriminatory power. To describe how much the choice of parameter values influences the discrimination of data points with low and high risk, we performed analysis of variance (ANOVA) between all 50 EPs based on their bootstrap replications. Thus, for each EP, 1000 bootstrap replicates of the SORS constituted an ANOVA group of estimates for a given parameter scenario. We calculated the F statistic in order to determine the degree of separation between the 50 groups of estimates with higher F values indicating higher degrees of separation. Therefore, the highest value of the F statistic within a given parameter variation analysis in this sense indicates the best result. The relative influence of the parameters on model results is estimated based on a normalization of the F statistic values. The algorithm used to implement deterministic and probabilistic analysis is shown in Fig. 2. Finally, we created heat maps based on the risk score estimates of the base case. For this purpose, the KDE values were again transferred back to the original map dimensions that were used in the pre-visualization step. To compare this base case risk map to an alternative visualization using a common geographical methodology, we estimated an inhomogeneous cluster point process with polynomial trend of degree two for positive and negative POIs that is designed to provide similar cluster structures compared to our KDE approach. Subsequently, we derived intensities for risk score plotting in a comparable way as it was done for the kernel density approach, i.e., via setting off the surfaces. We tested several point process types, such as “Thomas” and MatClust”, and chose the model with the best fit based on the Akaike Information Criterion [43]. The code for data download, data processing, and analysis of the scenarios defined above can be found in the supplementary information.

### Software

The spatial POIs were processed and analyzed using R version 4.0.2 [44]. For graphical visualization, R packages “ggplot2” [45], “graphics” [44], and “fields” [40] were used. We applied the “spatstat” [46] package to estimate Ripley’s K function and the bootstrap confidence bands around it. For KDEs, the packages “MASS” [47] and “sparr” [39] were chosen. Spatial data objects were built and handled via the packages “rgdal” [48], “geojsonR” [49], “prob” [50], and “spatstat” [46]. For interpolation and for the generation of risk score maps, the packages “fields” [40] and “gstat” [51] were used. To plot spatial objects on maps, we used the package “png” [52]. Finally, the DBSCAN algorithm was applied using the package “dbscan” [53].

## Results

### Spatial inhomogeneity and visual inspection of the study area

**Fig. 3:**
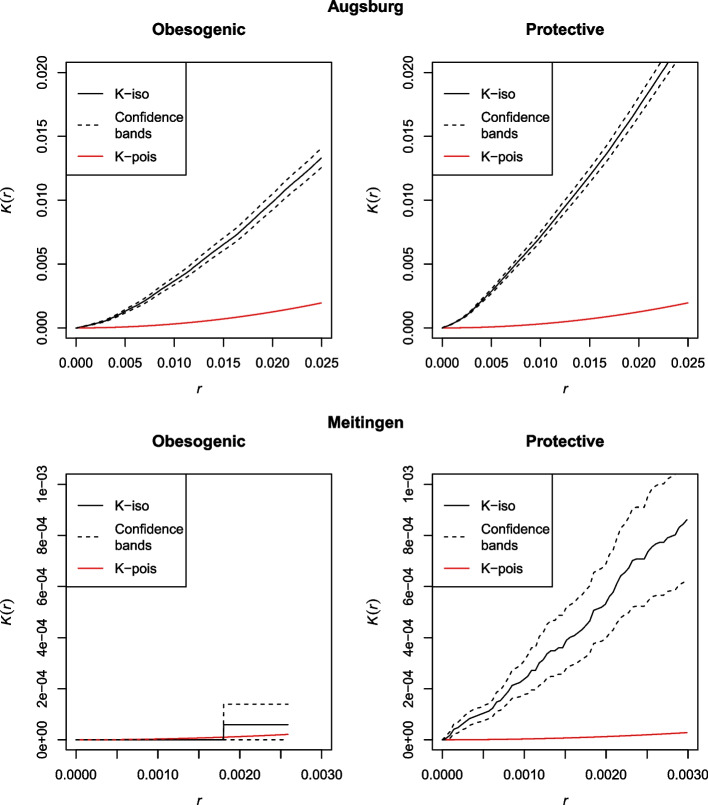
Spatial inhomogeneity measured via Ripley’s K function for Augsburg (top) and Meitingen (bottom), r = interpoint distance, K(r) = Ripley’s K function, iso = isotropic edge correction, pois = Poisson point process

The upper part of Fig. 3 shows estimates of Ripley’s K function for Augsburg, separately for the obesogenic and the protective POIs. The point pattern for Augsburg was significantly clustered, as the lower confidence bands of the K functions lay above the random Poisson processes for each interpoint distance r, which means that the actual number of POIs within a distance r was greater than the number of expected POIs under random distribution assumptions [31]. This underlined the importance of subsequent KDE analysis, as the spatial pattern was suitable for clustering tasks. Estimates of the obesogenic and the protective K functions for Meitingen also revealed significantly clustered patterns, as the lower confidence bands lay above the random pattern for all or at least for several interpoint distances r, which is shown in the bottom part of Fig. 3.

**Fig. 4:**
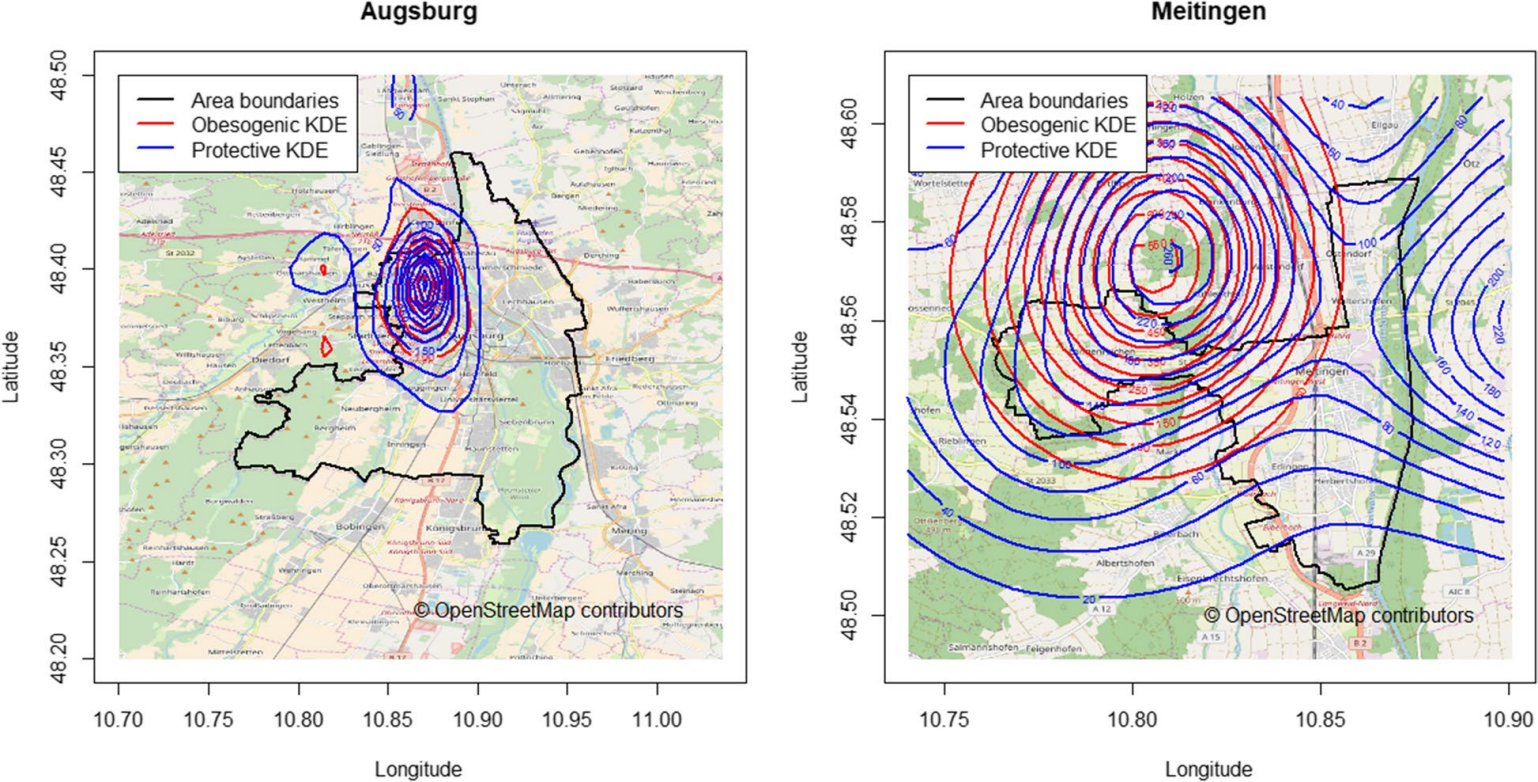
Contour lines of obesogenic and protective kernel densities in Augsburg (left) and Meitingen (right). Values of KDE are constant on a contour line with increasing values toward the respective KDE center

The separate obesogenic and protective risk surfaces are shown in Fig. 4 for Augsburg and Meitingen. The obesogenic and protective kernel densities for Augsburg accumulated within a region lying to the northwest within the city boundaries, whereas the eastern and the southern areas showed no dense region. In contrast, kernel densities for Meitingen showed several dense regions outside the town borders.

### Randomly drawn sample points

**Fig. 5:**
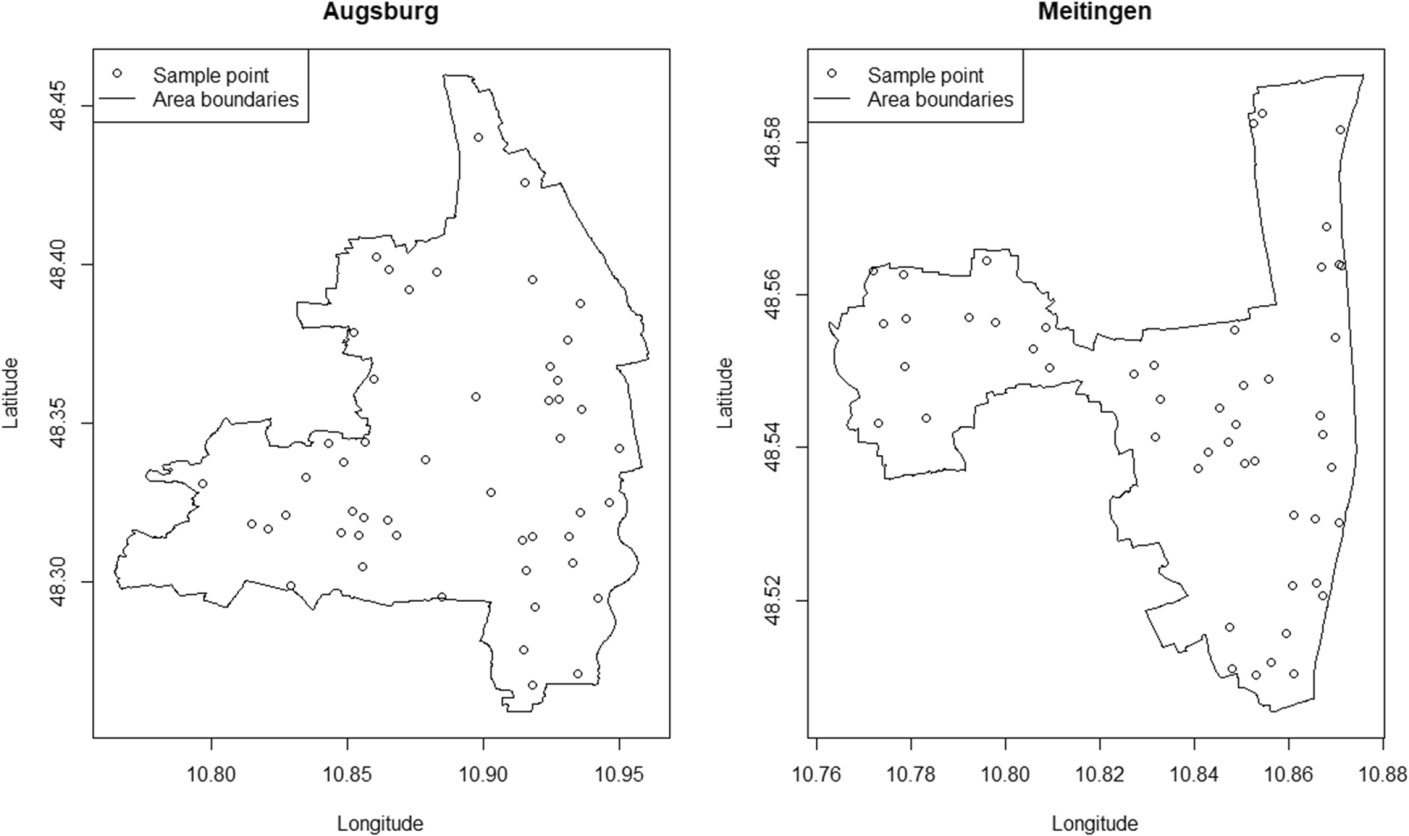
Final set of randomly chosen spatial data points for Augsburg (left, *N* = 50) and Meitingen (right, *N* = 50)

The final set of randomly chosen EPs for the evaluation of the risk score for Augsburg (*N* = 50) and Meitingen (N = 50) is presented in Fig. 5. As seen within the graphics, the sample points generated via the random drawing process inside the study areas widely covered the respective regions under consideration.

### Effect of parameter variation within KDE estimation process

Table 3 summarizes the values of the ANOVA F statistic for each scenario of base case and deterministic sensitivity analysis. The higher the F statistic for a given parameter variation, the higher the degree of separation between the sample point groups, and thus higher values were more preferable. Values of the F statistic for Augsburg and Meitingen increased with the amount of bandwidth for the first three scenarios. This trend continued for Meitingen with decreasing slope, whereas it showed a rather inverted u-shaped functional behavior for Augsburg. Regarding edge correction for Augsburg and Meitingen, increasing the study area to some degree led to the highest F statistic, but this effect was not permanently observed with increasing amounts of edge correction. For Augsburg, the second grid point scenario was preferred according to the ANOVA value, in contrast to Meitingen, for which the base case was preferred. Furthermore, the inverse distance weighting had the highest F value in Meitingen and Augsburg,. Finally, the second weighting scenario had a higher F value than the equal weighting scenario in Augsburg, whereas the opposite was seen for Meitingen. Overall, the bandwidth and the edge correction had the highest influence on the values of the F statistic.

**Table 3:** ANOVA F statistics for Augsburg and Meitingen

Augsburg		Meitingen	
Scenario^a^	F statistic^b^	Scenario^a^	F statistic^b^
BW1	871	BW1	1460
BW2	1095	BW2	3014
BW3 (BC)	1135	BW3 (BC)	4531
BW4	848	BW4	4779
BW5	657	BW5	4079
EC1 (BC)	1135	EC1 (BC)	4531
EC2	956	EC2	8372
EC3	724	EC3	9158
EC4	854	EC4	7047
EC5	1156	EC5	7912
GP1 (BC)	1135	GP1 (BC)	4531
GP2	1495	GP2	4293
GP3	1398	GP3	4530
INT1 (BC)	1135	INT1 (BC)	4531
INT2	1274	INT2	4628
INT3	1121	INT3	4306
INT4	875	INT4	6
WT1 (BC)	1135	WT1 (BC)	4531
WT2	1180	WT2	4335

*BC* Base case, *BW* Bandwidth, *EC* Edge correction, *GP* Grid points, *INT* Interpolation, *WT* Weighting

^a^ bandwidth, edge correction, and number of grid points presented in ascending order, i.e., with the first scenario describing the least amount of bandwidth, edge correction, and number of grid points respectively

^b^ The F statistic refers to the ANOVA F statistic between the groups of estimated data points at the 50 evaluation points for a given area (1000 data points at each evaluation point), calculated as follows: F = between-group variability/within-group variability; higher values of the F statistic reflect more preferable parameter values for a given scenario. The groups are generated based on the POI bootstrap replications

### Obesity risk score map

**Fig. 6:**
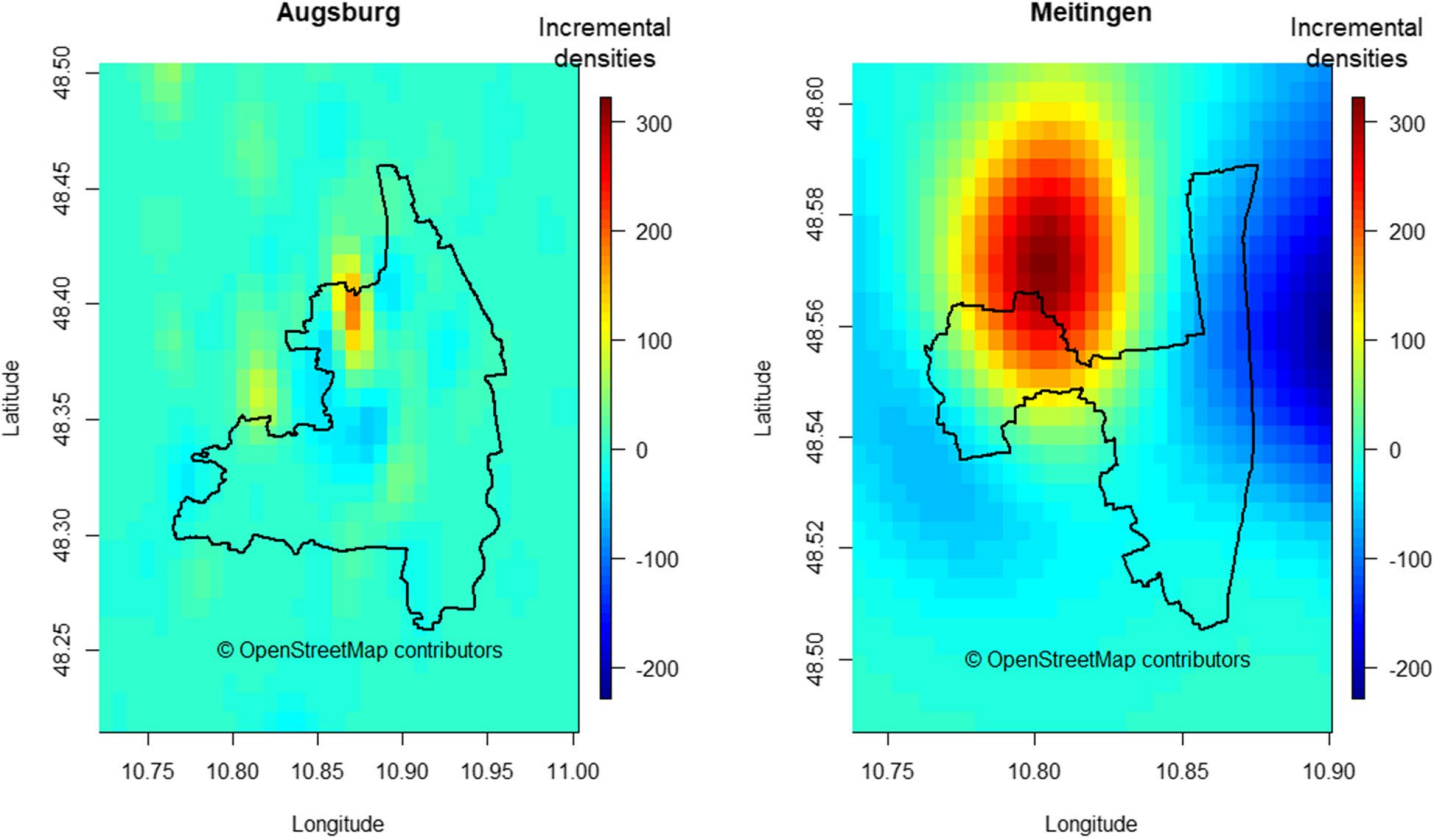
Risk score maps for Augsburg (left) and Meitingen (right) showing the base case with the following parameters: bandwidth: method of Terrell [38]; edge correction: area side length: 1.4 * side length of the exact box; number of grid points: 35 × 35; weighting scheme of the environmental factors: equal weighting with unity, interpolation here via the “image.plot” function

**Fig. 7:**
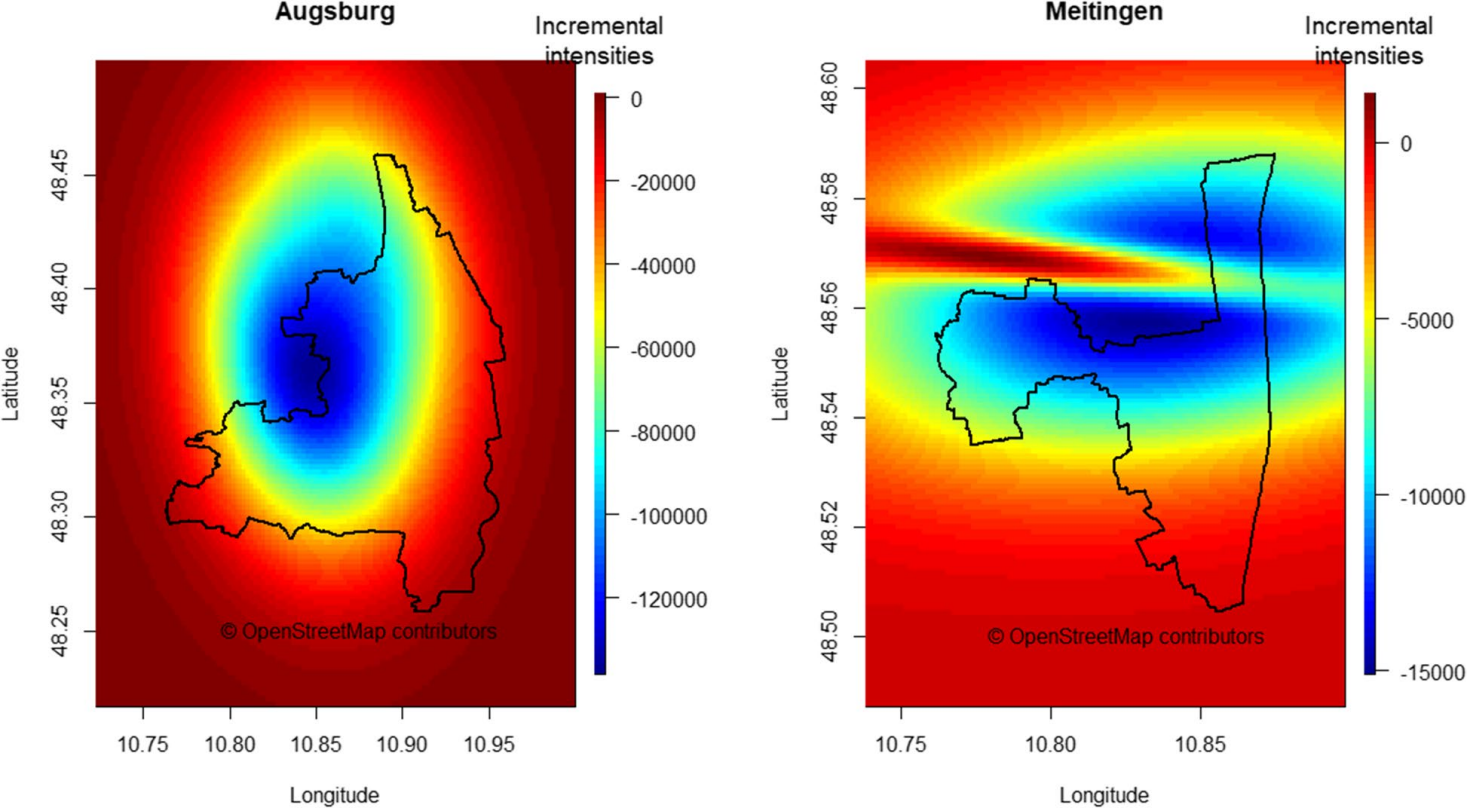
Risk Score map based on incremental intensities derived from inhomogeneous cluster point processes

Figure 6 depicts the risk score maps for Augsburg and Meitingen in which the five parameters bandwidth, edge correction, number of grid points, interpolation method and weighting, were set to their base case. The score values of the SORS are depicted as incremental densities resulting from subtracting the negative surface from the positive surface. The risk score map shows a composite picture of the separate estimates illustrated in Fig. 4. There was little heterogeneity in risk scores for the region of Augsburg except for a small area with higher obesogenic scores at the northwestern city boundary. The risk score map for Meitingen showed high risk scores for the northwestern part of the town as well as for the area north of the town borders. The risk score map based on point processes for comparison purposes is shown in Fig. 7. A region with a low obesity level is present in Figs. 6 and 7 for both study areas partly at similar places, however, obesity hotspots could only be derived from the SORS KDE plot in Fig. 6.

## Discussion

We developed the SORS based on KDE using freely available data from online geocoding services. We tested several parameters which could potentially influence the final score values. Our tests showed that the SORS depended on the choice of bandwidth and the amount of edge correction applied to the KDE; the latter, however, for only one of the two study areas. In contrast, the interpolation method, the numbers of grid points, and an alternative weighting scenario had a small influence on the results.

The SORS was calculated by taking the difference of the positive and the negative kernel density surface. We followed a similar approach to that in the work of Jones-Smith et al. [34]. They estimated correlations of their score with obesity. In contrast, the aim of our study was to investigate the effect of parameter variation on the robustness of our score. Furthermore, we covered an extensive list of obesogenic and protective environmental factors that expanded the approach of a food score to a more comprehensive measure, also including the physical activity environment. Some alternative approaches using KDE were based on the division of the kernel density surfaces [54]. A major drawback of these quotients is the issue with division by zero leading to values approaching infinity and thus leading to instability. Our approach to the SORS avoids this and also the need for some adjustments correcting for the instability.

Previous studies used various approaches to estimate risk scores based on kernel techniques, both in obesity-related research areas and elsewhere. Fitzpatrick and colleagues [55], for example, developed the keeping score based on KDE to characterize crime patterns, which has often been used by the police. Crime heat maps can be generated with this technique. This approach is based on the locations of past events instead of geolocated environmental factors, and the authors assumed that the pattern of these historic events would be maintained in the future.

Some studies created kernel density surfaces based on POIs and extracted density estimations from these surfaces in order to investigate the association with weight status. Rundle et al. (2009) analyzed the effects of environmental factors on body mass index (BMI). Results of KDE analysis concerning healthy and unhealthy food outlets were used to classify the neighborhood environment of each individual within the study based on a quintile approach [56]. Furthermore, walkability, land use mix, and population density were considered. These variables could not be implemented in our study based on the chosen POI approach with OSM data.

The five chosen SORS parameters, bandwidth, edge correction, grid points, interpolation, and weights, have also been investigated in the literature. Laraia et al. (2017) used a business software and ArcGIS to geocode the information from the study data [57]. As in our analysis, several bandwidths were tested within their KDE approach, which was found to be a sensitive model parameter. Similarly, we also found a fundamental influence of bandwidth on the results.

Effects at the edge of the study area were estimated in a simulation study concerning cluster models for food outlets [58]. Estimations at the boundaries were biased, and the authors came to the conclusion that edge effects should be corrected in studies considering measures of availability and accessibility. This underlined the importance of edge correction, which was also a major topic in our study. In addition, extending the study area has been proven to be a valuable edge correction method.

Finding the optimal number of grid points was also discussed in the literature. Some authors suggested that a choice between 100 and 500 grid points gives reasonable results [59]. In our analysis, we chose 25 × 25 points for the minimum bounding rectangle, i.e., 625 grid points, and chose some additional amount of edge correction for the base case. In addition, we performed some adjustments to preserve the distance between the grid points for the edge correction scenarios. In this case, the number of grid points was extended proportionally to the amount of edge correction applied, i.e., to the amount of study area extension. This made it possible to analyze grid point and edge effects separately. The choice of grid points in our base case and sensitivity analysis was chosen in accordance with default grid sizes implemented in KDE packages.

An inverse distance weighting method was applied in the past in KDE estimation regarding homicide locations as a parameter of area safety [60]. This method could be used to estimate effects at specific locations. We used such an inverse distance method in our model as an alternative to the automatic interpolation function of the base case. As a further common method, linear interpolation has been applied within the literature [61]. The “interp.surface” function applied to our SORS model was based on bilinear weights.

It was challenging to find a suitable weighting scheme applicable within our analysis. For the base case, we assumed that each factor has the same positive or negative weight, although this might look different in reality. Additionally, we tested an example from the literature [34]. We found that double weighting of supermarkets and physical activity facilities had little effect on the results. Owing to several possible weighting methods for spatial POIs, it is necessary to test further alternatives within future studies.

Finally, the SORS was graphically compared to a risk score that was derived from incremental intensities of inhomogeneous spatial point processes. Although the methodology applied here changed from KDE-based to intensity-based estimations, similar visual patterns could be derived from the two score approaches for protective patterns, which further underlines the robustness of our chosen algorithm.

### Implications of the SORS on obesity-related research and policy

The SORS is a helpful tool to understand the spatial distribution of health-related harmful environmental factors in relation to health-promoting environmental factors. Risk score maps allow for an overall intuitive view on summarized structures, which can be a valuable help in obesity-related research and also within policy. Although the actual use of those structures might look different in reality, it nevertheless gives a composite simplifying measure of the environment and can be further extended to a more comprehensive tool accounting for several health dimensions affecting individuals simultaneously.

### Strengths and limitations

Several strengths exist regarding our study. The automated processing of data and the automated testing of several important KDE parameters makes it possible to repeat the application of risk score estimation for other areas efficiently, given that the spatial data points and the shape files of the city or town boundaries have been downloaded before. This enables the user to describe, compare, and monitor (if done repeatedly) risk scores as well as the influence of relevant risk score parameters within several areas of interest, within other regions worldwide, and also on a larger geographic scale. For example, the analysis could be performed for a whole country in order to identify national inequalities regarding environmental obesity risks or to guide and prioritize prevention efforts that concentrate on the food and the physical activity environment. To achieve this on a regional scale, the data download area simply has to be increased to cover a larger area for the subsequent data download from OSM. The data files would be of a manageable size, as only a small number of features are important for this kind of analysis. For Augsburg, i.e., for the larger of our two study areas, the data file size was 8 MB. For larger areas, e.g., for Germany, other portals such as Geofabrik should be used. In this case, no query process is needed, and the data files are directly ready for download. The data size for Germany, for example, would be 3.1 gigabytes in this case [62]. Furthermore, using so-called planet OSM files, data disk space of around one terabyte (compressed 89 GB) or less is required [63].

We integrated uncertainty into our analysis by performing a spatial bootstrap. Subsequently, we used the samples directly for the evaluation of our method. This allowed us to assess the stability of the score values against POI variations and helped us to compare deterministic parameter scenarios based on the ANOVA F statistic. On the one hand, the impact of each parameter on score results could be assessed. In addition, the values of the F statistic could be used to find optimal parameter combinations for the SORS.

We checked the robustness of the score and repeated our analysis several times for a given area. Results were qualitatively equivalent, i.e., for each given parameter variation, the repeated analysis could be used to rank the scenarios in the same order.

However, there are also some limitations regarding the study. First, some of the environmental factors discovered during the literature search could not be implemented based on spatial POIs, especially complex constructs such as land use mix or walkability.

Second, the categorization of positive and negative obesogenic factors was based on data from pre-existing literature, and it is not known whether POIs categorized as “positive” or “negative” are really positively or negatively associated with obesogenic health (behavior). Further studies could compare the SORS with external data sources, such as walk scores in a given region, in order to test these associations [64].

As the content of OSM is generated by users, it is necessary to assess the data quality within validation studies. Within our previous work, we calculated sensitivity, specificity, and positive predictive values for OSM and compared the results with the corresponding values for Google Maps [18]. It became evident that both geocoding services performed adequately. OSM had higher positive predictive value but, in contrast, lower sensitivities than Google Maps.

## Conclusion

KDE has been proven to be a useful methodology in the development of an obesity risk score, predominantly on account of the nature of the continuous estimation approach enabling efficient generation of risk score maps. However, some parameters of KDE have a large effect on score results. Parameter optimization should therefore play a major role during score model development.

## Supplementary Information

**Additional file 1.** Complete list of chosen variables.

**Additional file 2.** Methodological details.

**Additional file 3.**

## Data Availability

The data generated for this study were downloaded from OSM using the tool Overpass turbo [https://overpass-turbo.eu/. Accessed 11 Aug 2022], and the exemplary code is included in this published article.

## Abbreviations

ANOVA: Analysis of variances
API: Application Programming Interface
BC: Base case
BS: Bootstrap sample
BW: Bandwidth
EC: Edge correction
EP: Evaluation point
GEDA: (GEDA, German Health Update)
GIS: Geographic information systems
GP: Grid points
INT: Interpolation
JSON: JavaScript Object Notation
KDE: Kernel density estimation
OSM: OpenStreetMap
POI: Point of interest
SORS: Spatial obesity risk score
WT: Weighting
