# Supplementary material for: A spatial obesity risk score for describing the obesogenic environment using kernel density estimation: development and parameter variation
Source: BMC Med Res Methodol. 2023 Mar 17;23:65. doi: 10.1186/s12874-023-01883-y (PMC10021981; doi:10.1186/s12874-023-01883-y)
Supplement: Supplementary file 1 — Additional file 1. Complete list of chosen variables. [file 12874_2023_1883_MOESM1_ESM.docx]

Additional File 1: Complete list of chosen variables

Table A1: Variables separated by category

| Category | Variables | | |
| --- | --- | --- | --- |
| Amenity | bar § | bbq * | bicycle_parking § |
|  | bicycle_rental § | biergarten § | boat_rental § |
|  | boat_sharing § | bus_station * | car_rental * |
|  | car_sharing * | cafe § | clinic § |
|  | college § | dentist § | dive_centre § |
|  | doctors § | dojo § | fast_food * |
|  | fire_station § | food_court § | fountain § |
|  | hospital § | hunting_stand § | ice_cream * |
|  | kneipp_water_cure § | language_school § | library § |
|  | marketplace § | nursing_home § | pharmacy § |
|  | police § | prison § | ranger_station § |
|  | restaurant § | school § | taxi * |
|  | university § |  |  |
| Leisure | beach_resort § | dance § | dog_park § |
|  | fishing § | fitness_centre § | fitness_station § |
|  | garden § | golf_course § | ice_rink § |
|  | miniature_golf § | nature_reserve § | park § |
|  | pitch § | playground § | sports_centre § |
|  | stadium § | summer_camp § | swimming_area § |
|  | swimming_pool § | track § | water_park § |
| Land use | allotments § | farmland § | farmyard § |
|  | forest § | grass § | greenfield § |
|  | greenhouse_horticulture § | meadow § | orchard § |
|  | plant_nursery § | recreation_ground § | village_green § |
|  | vineyard § |  |  |
| Natural | fell § | grassland § | heath § |
|  | scrub § | tree § | tree_row § |
|  | water § | wood § |  |
| Shop | bakery § | beverages § | bicycle § |
|  | butcher § | cheese § | coffee § |
|  | convenience * | dairy § | deli § |
|  | farm § | garden_centre § | garden_furniture § |
|  | greengrocer § | fishing § | free_flying § |
|  | hunting § | ice_cream * | medical_supply § |
|  | nutrition_supplements § | outdoor § | pasta § |
|  | pastry * | scuba_diving § | seafood § |
|  | spices § | sports § | supermarket § |
|  | swimming_pool § | tea § | wine § |
| Vending | bicycle_tube § | bread § | chemist § |
|  | coffee § | first_aid § | fishing_tackle § |
|  | food § | ice_cream * | milk § |
|  | sweets * |  |  |
| Other variables (category) | aerodrome (aeroway) * | aquarium (tourism) § | bakery (craft) § |
|  | bus_stop (highway) * | camp_site (tourism) § | caterer (craft) § |
|  | halt (railway) * | helipad (aeroway) * | heliport (aeroway) * |
|  | national_park (boundary) § | picnic_site (tourism) § | sport, whole category § |
|  | rest_area (highway) § | station (railway) * | theme_park (tourism) § |
|  | therapist (office) § | tram_stop (railway) * | zoo (tourism) § |

* = obesogenic, § = protective
