# Supplementary material for: A spatial obesity risk score for describing the obesogenic environment using kernel density estimation: development and parameter variation
Source: BMC Med Res Methodol. 2023 Mar 17;23:65. doi: 10.1186/s12874-023-01883-y (PMC10021981; doi:10.1186/s12874-023-01883-y)
Supplement: Supplementary file 2 — Additional file 2. Methodological details. [file 12874_2023_1883_MOESM2_ESM.docx]

Additional File 2: Methodological details

Points of Interest download

Spatial points of interest (POIs) were downloaded as GeoJSON-Files, a file format in JavaScript Object Notation (JSON) style containing geographic data based on a key value structure, from OSM. This was executed via the online data filtering tool Overpass turbo, which enables access to the Overpass Application Programming Interface (API) of OSM [1]. Queries formulated via specific API query language were executed with this tool [2]. The tool was also used to visualize the spatial data points on an embedded map on the web page.

A rectangular area, a so-called bounding box, around each study region was chosen for the download of POIs. The size of this area was chosen large enough to allow for edge correction of the smaller contained study area in which variation in the SORS parameters was investigated.

Details regarding the coordinate adaptation and the synthetic origin

Within online geocoding services, the actual length of the longitudinal coordinate depends on the latitude to conserve conformal projections [3]. In order to correct this bias for kernel density estimation, we transformed the coordinates to adapt the length of one unit in the latitudinal direction to one unit in the longitudinal direction, i.e., we multiplied the longitudinal coordinate by a factor of 0.64 applicable within regions lying around 48 latitudinal degrees. To simplify this process, we introduced a synthetic origin of coordinates lying to the south-west of both study areas. In order to preserve original map dimensions for graphical visualization of the risk score estimates, we used the corrected risk score estimates and shifted them back to the original map dimension.

Details regarding bus stop clustering

We decided to perform a previous clustering on bus stops, as this variable seemed to be overrepresented compared with the expected impact of a single POI. Therefore, we implemented density-based spatial clustering (DBSCAN) in order to find dense regions of bus stops [4]. For risk score estimation, the obesogenic spatial POIs containing the bus stops and the protective POIs were handled altogether in two separate data layers. In order to reduce the number of bus stops into several dense clusters of bus stops, we integrated the longitude–latitude pairs of all bus stops into a data frame and applied the DBSCAN algorithm. Subsequently, we replaced the bus stop data points of the obesogenic data layer with the centroids of the discovered bus stop clusters. We assumed a cluster to be dense if at least five POIs were discovered within an epsilon neighborhood of 50 meters.

Incremental sampling process for edge correction

We extended the area and simultaneously the number of data points around the study area with increasing amount of edge correction. Therefore, we chose an incremental sampling procedure analogous to the increase in grid points across these edge correction scenarios. This improved comparability across the parameter variation. As a first step, a sample was generated for the smallest area, i.e., the base case edge correction. For each next edge correction scenario, the inner sample points from the previous sample step were maintained, and the remaining sample points were generated from the additional POIs of the gradually extended KDE estimation area. In all, this led to the generation of 1,000 incremental bootstrap samples for the obesogenic POIs and 1,000 incremental samples for the protective POIs.

References

1. Overpass turbo. https://overpass-turbo.eu/. Accessed 11 Aug 2021.

2. Overpass API/Language Guide. https://wiki.openstreetmap.org/wiki/Overpass_API/Language_Guide. Accessed 11 Aug 2021.

3. Genauigkeit von Koordinaten. https://wiki.openstreetmap.org/wiki/DE:Genauigkeit_von_Koordinaten. Accessed 25 Aug 2021.

4. Ester M, Kriegel H-P, Sander J, Xu X. A density-based algorithm for discovering clusters in large spatial databases with noise. 1996. p. 226–31.
